# Responses of Fine Roots at Different Soil Depths to Different Thinning Intensities in a Secondary Forest in the Qinling Mountains, China

**DOI:** 10.3390/biology11030351

**Published:** 2022-02-22

**Authors:** Yue Pang, Jing Tian, Hang Yang, Kai Zhang, Dexiang Wang

**Affiliations:** College of Forestry, Northwest A&F University, Yangling, Xianyang 712100, China; pangyue1109@163.com (Y.P.); tianjing842@163.com (J.T.); yhang07@126.com (H.Y.); zhangkai260019@163.com (K.Z.)

**Keywords:** fine-root characteristics, fine roots with different diameters, deeper soil depth, whole-tree harvesting, secondary forest

## Abstract

**Simple Summary:**

Fine roots of different sizes make critical contributions to carbon stocks and terrestrial productivity, but the changed characteristics of fine roots with different diameters at different soil depths under thinning disturbances remain poorly understood. In our study, we aimed to elucidate the response characteristics of fine roots with different diameters to thinning intensities at different soil depths, and to explore the driving mechanism of the change in the fine-root characteristics. We found that higher thinning intensities negatively affected 0.5–1 mm and 1–2 mm fine-root biomass, while the <0.5 mm fine-root characteristics fluctuated with increasing thinning intensities. Our results suggest that the thinning intensity exhibits varied influential mechanisms on the changed characteristics of <0.5 mm fine roots and thicker fine roots (0.5–2 mm). Collectively, our findings provide important insights into the effects of forest management on changes in fine-root characteristics, and supplement meaningful data on fine-root productivity to improve the parameterization of future ecological models.

**Abstract:**

Fine roots make critical contributions to carbon stocks and terrestrial productivity, and fine roots with different diameters exhibit functional heterogeneity. However, the changed characteristics of fine roots with different diameters at different soil depths following thinning disturbances are poorly understood. We investigated the biomass, production, mortality and turnover rate of <0.5 mm, 0.5–1 mm, and 1–2 mm fine roots at 0–20 cm, 20–40 cm, and 40–60 cm soil depths under five thinning intensities (0%, 15%, 30%, 45%, and 60%) in a secondary forest in the Qinling Mountains. The biomass, production and turnover rate of <0.5 mm fine roots fluctuated with increasing thinning intensities, while the 0.5–1 mm and 1–2 mm fine-root biomass significantly decreased. The thinning intensities had no effects on the fine-root necromass or mortality. The change in the fine-root characteristics in deeper soils was more sensitive to the thinning intensities. The principal component analysis results showed that increased <0.5 mm fine-root biomass and production resulted from increased shrub and herb diversity and biomass and decreased soil nutrient availability, stand volume, and litter biomass, whereas the 0.5–1 mm and 1–2 mm fine-root biomass showed the opposite trends and changes. Our results suggest that different thinning intensities exhibit varied influential mechanisms on the changed characteristics of fine roots with different diameters.

## 1. Introduction

Thinning operations, a key forest management practice, are widely employed to maintain secondary forest ecosystem stability and promote stand productivity [1]. Thinning decreases stand density, regulates stand structure, reduces self-thinning mortality and wildfire risk, accelerates the rapid growth of remaining trees, increases resistance to natural disasters, stimulates ecosystem nutrient cycling processes, and improves forest health [2,3,4,5]. Additionally, reasonable thinning can also improve the stand environment and promote the regeneration of understory vegetation, and transform energy consumption caused by competition into effective productivity [6,7]. Nevertheless, little is known about the impact of thinning intensity on belowground productivity, although the belowground parts of vegetation make critical contributions to terrestrial productivity and carbon stock [3]. This is especially true for fine roots (Ø ≤ 2 mm). Fine roots account for less than 5% of all forest biomass but for 30–50% of total net primary production [8], and for approximately 50–70% of the soil C flux in forest ecosystems [9]. Unlike the sampling of other aboveground organic parts, the sampling of plant roots at the stand level is destructive, laborious and technically challenging, leading to incomplete estimations and understanding of fine-root processes [10].

Abiotic and biotic factors are the main drivers of change in fine-root characteristics (biomass, production, necromass, mortality and the turnover rate) [11,12]. Thinning treatments can directly modify the physical structure of trees and understory vegetation, and indirectly influence belowground environmental conditions (e.g., soil fertility and microclimate), potentially impacting changes in fine-root characteristics [13,14]. However, a few studies have reported contrasting results regarding thinning. For instance, some studies have shown that the biomass and production of fine roots generally increase following thinning due to improved soil nutrient ability and soil moisture levels [15,16]. In contrast, fine-root biomass and production responses to thinning have been reported in other studies, including negative and negligible responses [17,18]. These inconsistencies may result from the variations in sampling heterogeneity, plant species composition and climatic conditions. Undoubtedly, the exploration of the underlying mechanisms of change in fine-root characteristics’ responses to thinning intensity will provide a theoretical basis for the further understanding of productivity and nutrient cycling in terrestrial ecosystems.

Fine roots are traditionally defined as roots with a diameter of <2 mm, though this definition has recently been debated [19]. Even in this small-diameter category (Ø ≤ 2 mm), fine roots of different sizes exhibit functional heterogeneity due to their different physiological activities [20,21]. Fine roots of <0.5 mm (very fine) are mainly responsible for the acquisition and absorption of resources in the soil, and 0.5–2 mm fine roots (thicker) are responsible for resource transfer and storage [22,23]. Very fine roots are typically more dynamic than thicker roots, and are extremely sensitive to environmental change, as the ratio of nonstructural to structural mass is much higher in smaller roots [15,24]. For instance, the responses of fine roots to soil nutrient and water changes are diameter dependent [25,26]. Furthermore, Ma et al. (2013) reported that very fine roots have a higher turnover rate and productivity levels than thicker roots following thinning.

Plants may also adjust their root systems to use resources at different soil depths [27]. When they are stimulated by more resource competition in the surface soil, plants can adjust their roots at deep soil depths to obtain more soil resources [10]. When competition for surface soil resources decreases, the exploration of deep-layer soil resources is also reduced, and the plant root system will again shift to the utilization of surface resources [28]. Thinning alters the original vegetation configuration, resulting in a change in resource competition among species that in turn leads to varied resource use strategies being employed by fine roots at different soil depths [29]. Light- and high-intensity thinning slightly or significantly reduce resource competition, leading deep-soil resource exploration to decrease [16]. Under a suitable thinning treatment, understory species diversity increases and resource competition intensifies, and fine roots gradually explore deep-soil resources, resulting in increased fine-root biomass and production at deeper soil depths [14]. It can be seen that there may be larger fluctuations in the characteristics of fine roots at the deep soil under disturbance. Thus, collecting samples at deeper soil depths is needed in order to fully quantify the change in fine-root characteristics and spatial distribution variability [30], and to clarify the resource acquisition strategies of fine roots under different thinning treatments.

In the present study, we determined the biomass, necromass, production, mortality, and turnover rate characteristics of <0.5 mm, 0.5–1 mm, and 1–2 mm fine roots at 0–20 cm, 20–40 cm and 40–60 cm soil depths under five thinning intensities (0%, 15%, 30%, 45%, and 60% of the stand volume removed) in secondary forests. Our hypotheses are as follows: (a) changes in very fine-root characteristics are more sensitive to thinning intensities than thicker fine roots due to their greater responsiveness to changing environments; (b) fine-root necromass and mortality increase when thinning intensities increase, as cutting trees would lead to the death of root systems [31,32]; and (c) changes in fine-root characteristics in deep soils are more sensitive to thinning intensities than those in shallow soils, and exhibit fluctuations because plants must flexibly adjust the root system to use resources at deep soil layers under the changing pressures of soil resource competition. Furthermore, in order to understand the driving effects of abiotic and biotic conditions on the biomass, production, necromass, mortality and turnover rate of fine roots, we explored the linkages between fine-root characteristics, soil properties and stand characteristics.

## 2. Materials and Methods

### 2.1. Study Area

The experiment was implemented at the Qinling National Forest Ecosystem Research Station (33°18′–33°28′ N, 108°21′–108°39′ E) located on the Huoditang Experimental Forest Farm of Northwest A&F University, in Ningshaan County, Shaanxi Province, China. This study area covers an area of 22.25 square kilometers, and has a subtropical humid montane climate with altitudes ranging from 1900 to 2200 m and a mean slope of approximately 35°. Across the site, Cambisols, Umbrisols and Podzols (FAO) are the dominant soil types, reaching a mean soil depth of 50 cm [33]. The annual average temperature and humidity are approximately 10.5 °C and 77%, respectively. The annual mean precipitation level is 1000 mm, with 50% occurring from July to September. Local plants have a 177-day growth period, while the average frost-free period of the study area is approximately 199 days [34]. During the 1960s and 1970s, forests around the Qinling National Forest Ecosystem Research Station underwent extensive logging. After decades of regeneration, the forest coverage rate reached 93.8%, and secondary growth dominated the area. The dominant tree species are *Quercus aliena* var. *acutiserrata*, *Quercus variabilis*, *Pinus armandii*, *Betula albosinensis*, *Picea asperata*, and *Populus davidiana*. Shrubs (i.e., *Lonicera tragophylla*, *Cerasus stipulacea*, and *Symplocos paniculata*) and herbs (i.e., *Lysimachia christinae*, *Rubus parvifolius*, *Saussurea mutabilis*, and *Rubia cordifolia*) occupy the understory space [35,36,37].

### 2.2. Experimental Design and Treatments

**Figure 1:**
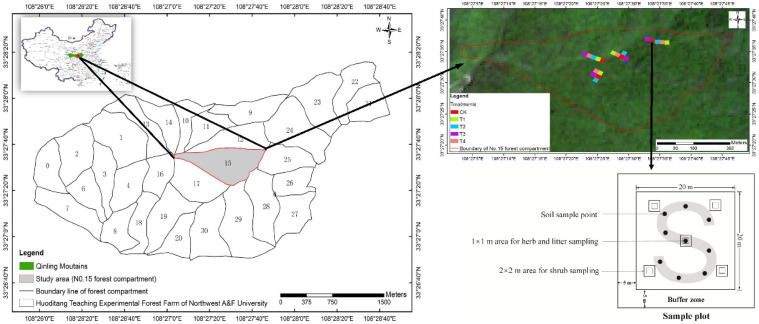
Location of the study area and the layout of the experimental plots. CK, T1, T2, T3, and T4 represent 0%, 15%, 30%, 45% and 60% thinning intensities, respectively.

For the purpose of promoting high-quality trees, forest farm staff cut down disturbing trees in the secondary forests according to the target tree operation system. Whole-tree harvesting was used during the felling, which removed most of the aboveground tree parts (stems, needles, branches, and twigs) to a greater extent than conventional stem-only harvesting [38,39]. These thinning treatments in secondary forest stands (dominated by *Pinus armandii*, *Betula albosinensis*, and *Picea asperata*) were carried out from July to September 2013. All of the selected plots were of the same stand age (35 years), occupied a similar topography, and had no history of fertilization. A randomized complete block design was used in the study. Five 20 × 20 m plots were randomly installed within each secondary forest block. Based on the target tree operation system, the thinning intensities included: (1) no thinning (CK), (2) the 15% removal of the stand volume (T1), (3) the 30% removal of the stand volume (T2), (4) the 45% removal of the stand volume (T3), and (5) the 60% removal of the stand volume (T4). Given that the study area had undergone large-scale logging, the forest community is now at a young stage. Therefore, combined with the Chinese forestry industry standard, a buffer zone of 5 m was set to avoid potential edge effects. All of the harvesting debris was removed from the plots. Each of the five thinning treatments was replicated into four blocks, totaling 20 sampling plots (five thinning intensities × four blocks). The layout of the experimental design is shown in Figure 1 (including the blocks and plots).

### 2.3. Soil, Litter and Vegetation Survey

The soil sampling was conducted at three soil depths (0–20 cm, 20–40 cm and 40–60 cm) using a soil auger (40-mm diameter) in August 2018. We collected nine replicate soil samples following an “S”-shaped pattern at three depths in each plot (Figure 1). Then, the collected soil samples were fully homogenized from the same depth to form a composite soil sample. In total, 60 composite soil samples (5 treatments × 4 blocks × 3 depths) were collected. Plant and fauna residues were manually removed, and the soil was then passed through a 2-mm screen. The soil samples were then divided into two portions: the first part was air-dried to measure the soil organic carbon, pH, available nitrogen, available phosphorus and available potassium. The second portion was used to measure the water content after oven-drying at 105 °C for 48 h. Soil samples of three duplicates were collected at depths of 0–20 cm, 20–40 cm and 40–60 cm by volumetric rings (100 cm^3^) after continuously sunny conditions in order to measure the soil’s bulk density [34,40].

The tree height (H) and diameter at breast height (DBH ≥ 5 cm, 1.3 m) in each plot were measured. The understory species diversity was investigated in five shrub subplots (2 × 2 m) and five herb subplots (1 × 1 m) established along the diagonals in each plot. Whole-plant sampling techniques were used to determine the shrub and herb biomass [33]. For the litter sampling, all of the organic material (the undecomposed and decomposed parts on the ground) in five 1 × 1 m subplots were collected. Herb and litter subplots were located on larger shrub subplots, and all of the vegetation surveys were carried out in August at peak vegetation coverage, as previously described [41].

### 2.4. Fine-Root Sampling

The sequential soil coring method was used to collect fine-root biomass, production, mortality and turnover rate data using a previously described method [42]. Because the change in fine-root characteristics exhibits strong seasonal variations, fine roots were sampled throughout the year [43]. Furthermore, we expanded the soil depth interval to 0–60 cm based on the average soil layer thickness in the study area.

In each sampling plot, we randomly collected eight soil cores (90-mm inner diameter) over the first three days of September (autumn) and November (winter) 2018, and of April (spring) and June (summer) 2019 (no samples were collected from December to March because low temperatures had caused the soil layer to freeze). We collected samples at depths of 0–20, 20–40 and 40–60 cm using a soil auger, producing 480 (5 treatments × 4 blocks × 8 cores × 3 depths) samples seasonally and 1920 (480 samples × 4 seasons) samples for the four seasons of sampling used for the laboratory analysis. The root samples collected at each soil depth were thoroughly mixed to form one fine-root sample. In total, 240 root samples (5 treatments × 4 blocks × 3 depths × 4 seasons) were collected over the four seasons.

The composite fine-root samples were transported to the laboratory in an icebox. In order to separate the roots from the soil, we first soaked the fine-root samples in water. Then, three diameter classes of fine roots (<0.5, 0.5–1, and 1–2 mm, determined using electronic calipers) were carefully washed and sorted into living and dead groups according to their status using the method described by Brassard et al. (2013) [44]. Live roots were classified as having a pale exterior, as elastic and flexible, and as being free of decay, with a whitish cortex, while dead roots were brown or black in color, and were inflexible. Finally, all of the live and dead fine roots of the three diameters were oven-dried at 65 °C to a constant mass.

### 2.5. Chemical and Biochemical Analyses

All of the soil chemical indicators were determined following a previously described method [40]. The soil’s organic carbon content of the soils was measured using the K_2_Cr_2_O_7_ oxidation method. The soil’s available nitrogen was identified by alkaline hydrolysis diffusion, and the available phosphorus was measured by colorimetry after extraction with NaHCO_3_. The soil’s available potassium was extracted in ammonium acetate (pH 7.0) and identified on a flame photometer. The soil pH was determined in a 1:2.5 soil:water suspension. The soil’s bulk density was obtained by calculating the ratio of the soil’s mass to the total volume (g·cm^−3^) after oven drying at 105 °C to a constant weight [45].

### 2.6. Data Calculation and Analysis

The fine-root biomass (g m^−2^) and necromass (g m^−2^) were calculated for each sampling season in each plot by summing the dry weight of the live and dead fine roots in each soil core. The fine-root production (g m^−2^ year^−1^) and mortality (g m^−2^ year^−1^) were determined using a simplified decision matrix method (Table 1) [14]. The fine-root turnover rate (year^−1^) was defined as the ratio of annual fine-root production (g m^−2^ year^−1^) to the mean biomass (g m^−2^) of fine roots over a year [46].

In the present study, the biomass characteristics of fine roots were repeatedly measured across sampling seasons according to the soil depth within each plot. Therefore, we performed a linear mixed-model analysis with three fixed effects (thinning intensity (T), sampling season (S), and soil depth (D)) and random effects (plot and block), as described by Feng et al. (2018) and Wang et al. (2019):

(1) $$Y_{ijkl}=T_{i}+S_{j\left(l\right)}+D_{k\left(l\right)}+T_{i}\times S_{j\left(l\right)}+T_{i}\times D_{k\left(l\right)}+S_{j\left(l\right)}\times D_{k\left(l\right)}+T_{i}\times S_{j\left(l\right)}\times D_{k\left(l\right)}+\pi_{l}$$

where *Y_ijkl_* is the fine-root biomass, necromass, or total mass (g m^−2^); *T_i_* (*i* = 0, 15, 30, 45, 60) is the thinning intensity; *S*_*j*(*l*)_ is the sample season (i.e., autumn, winter, spring, or summer); *D*_*k*(*l*)_ is the soil depth (0–20 cm, 20–40 cm, and 40–60 cm); and *π_l_* is a random plot or block effect (*l* = 1, 2, …, 20).

Under different thinning intensities, the changes in the fine-root characteristics were highly variable at different soil layers. Thus, the fine-root characteristic percentage change (compared to CK) of the average for all of the thinning intensities at a certain soil depth could be seen as an indicator for the evaluation of the fine-root characteristic responses at different soil depths to thinning intervention. A higher percentage change of one soil depth indicates a more sensitive response to thinning intensities, which was calculated as follows:

(2) $$C_{p}=\frac{\sum_{n=1}^{4}\left(\frac{\left|C_{T_{n}}-C_{CK}\right|}{C_{CK}}\right)}{4}\times 100\%$$

where *C_p_* denotes the fine-root biomass, necromass, total mass, production, mortality or turnover rate percentage change value; *T_n_* (*n* = 1, 2, 3, 4) is the thinning intensity; *C_Tn_* and *C_CK_* are the fine-root characteristic mean values for the thinning and control conditions, respectively; and |*C_Tn_* − *C_CK_*| represents the absolute value of *C_Tn_* − *C_CK_*.

The effects of thinning intensities on the soil properties (water content, bulk density, soil organic carbon, available nitrogen, available phosphorus, available potassium and pH) and stand characteristics (tree density, height, DBH and volume, understory vegetation biomass and species diversity index, and litter biomass) were also tested using a linear mixed-effects model ANOVA. For all of the models, the significance of fixed effects was assessed using Satterthwaite approximations for degrees of freedom. When the fixed effects or interactions were significant, the least square means differences test was performed for multiple comparisons (main effect or simple effect analysis). The statistical value F was used to evaluate the sensitivity differences of three diameter classes of fine roots to thinning intensities. The linear mixed-effects model was obtained with the ‘lmerTest’ and ‘lme4’ packages [47,48]. Multiple comparisons were drawn using the ‘emmeans’ package. Principal component analysis (PCA) was performed to determine the relationships between the fine-root characteristics, stand characteristics and soil properties, using the ‘FactoMineR’ package [49]. All of the analyses were implemented using R for Windows statistical software, version 4.1.1 [50].

## 3. Results

### 3.1. Stand and Soil Properties

The stand and soil properties changed in different ways following the thinning treatments (Table 2). The stand volume and litter biomass significantly decreased with increasing thinning intensities (*p* < 0.05), whereas the biomass and Shannon–Wiener index of the shrubs and herbs showed the opposite trend (*p* < 0.05).

The soil’s organic carbon, available nitrogen, available phosphorus, available potassium, water content and pH values decreased with increasing soil depths, while the soil’s bulk density increased. T2, T3 and T4 had significantly lower soil organic carbon, available nitrogen, available phosphorus and available potassium values than their CK values, decreasing by 47.1–72.3%, 23.2–77.6%, 18.7–61.8 and 6.3–53.6% (*p* < 0.05), respectively. Thinning had no significant effects on the soil’s water content, bulk density or pH (*p* > 0.05) (except for the pH at 40–60 cm) (Table 2).

### 3.2. Fine-Root Biomass, Necromass, and Total Mass of Different Diameter Classes

**Figure 2:**
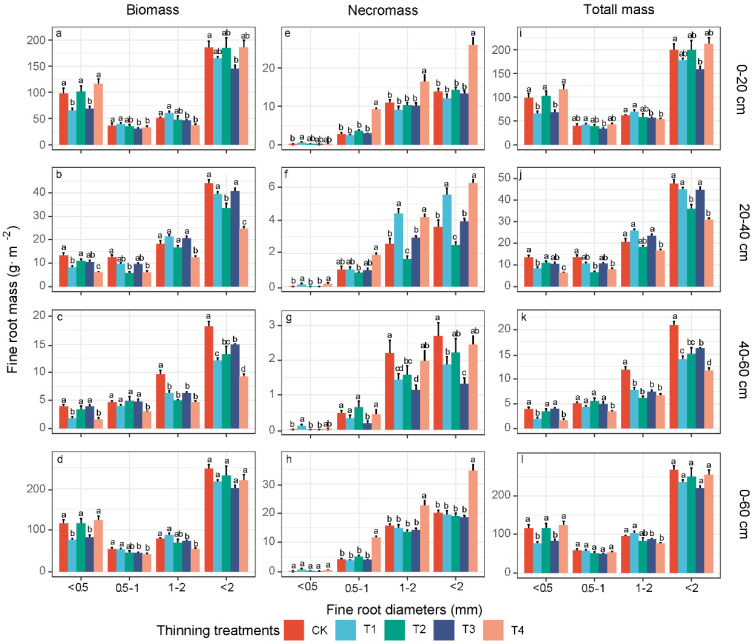
The effects of thinning intensities on the fine-root biomass, necromass, and total mass (average over four seasons) among different diameter classes at different soil depths (**a**–**c**,**e**–**g**,**i**–**k**), and the entire soil profile (**d**,**h**,**l**). The values are the mean of four replicates ± SE. CK, T1, T2, T3, and T4 represent 0%, 15%, 30%, 45% and 60% thinning intensities, respectively. Different lowercase letters (a, b, c, d) indicate significant differences for the same diameter class fine roots among the thinning intensities (*p* < 0.05).

The whole fine-root system (Ø ≤ 2 mm) biomass (F = 2.11, *p* > 0.05), necromass and total mass (F = 2.33, *p* > 0.05) values of the total soil depth (0–60 cm), on average, did not differ among the treatments, except for the significant increase in necromass occurring in T4 (F = 25.31, *p* < 0.01) (Figure 2d,h,l and Table 3). The very fine-root (<0.5 mm) biomass and total mass fluctuated with the increasing thinning intensity. The very-fine root biomass (F = 10.91, *p* < 0.01) and total mass (F = 10.62, *p* < 0.01) in T1 and T3 were significantly lower than those in CK, while those in T2 and T4 exceeded those in CK (although not significantly) (Figure 2d,l and Table 3). The 0.5–1 mm (F = 4.42, *p* < 0.05) and 1–2 mm (F = 9.01, *p* < 0.01) fine-root biomass, and the 1–2 mm (F = 4.15, *p* < 0.05) total mass decreased with the thinning intensity, showing a significant difference at T3 and T4 (Figure 2d,l and Table 3). The 0.5–1 mm and 1–2 mm fine-root necromass mirrored the overall fine-root system necromass characteristics, whereas very fine-root necromass was rarely observed (Figure 2h).

The fine-root biomass, necromass, and total mass decreased with the soil depth, and the values in the topsoil (0–20 cm) accounted for more than 70% of the total values for all of the soil depths (Figure 2). The biomass and the total mass of the very fine roots were mainly distributed in the topsoil, and accounted for approximately 50% of the values in the topsoil and 40% of the total values. The biomass and total mass of 0.5–1 mm and 1–2 mm roots dominated the deep soil depths, while the necromass of 0.5–1 mm and 1–2 mm roots was dominant at all of the soil depths (Figure 2). The very fine-root biomass and total mass were more sensitive to the thinning intensities than those of other size classes across all of the soil depths (Table 3 and Figure 2). The fine-root biomass, necromass, and total mass at the middle and deep soil depths exhibited greater percentage changes than those in the topsoil following thinning, and these characteristics in the deep soil increased again in T3 and T2 (Figure 2 and Table 4).

**Figure 3:**
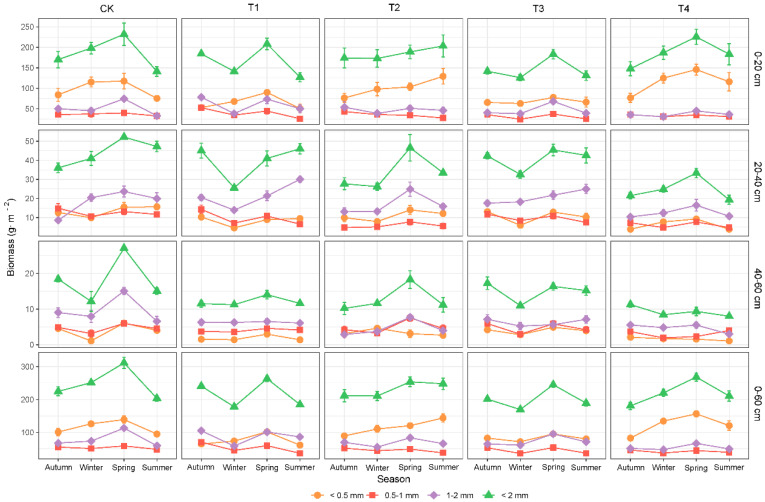
The effects of the season on the fine-root biomass (live) among different diameter classes in different soil depths, and the entire soil profile. The values are the mean of four replicates ± SE. CK, T1, T2, T3, and T4 represent 0%, 15%, 30%, 45% and 60% thinning intensities, respectively.

The fine-root biomass, necromass, and total mass exhibited strong seasonal variations within the sampling year (Figure 3, Figures S1 and S2 and Table S1). The fine-root biomass and total mass peaked in spring, as determined by the very fine-root biomass levels (Figure 3 and Figure S2). The fine-root necromass peaked in the autumn, as did the 0.5–1 mm and 1–2 mm fine-root necromass (Figure S1).

### 3.3. Fine-Root Production, Mortality and Turnover Rate in Different Diameter Classes

**Figure 4:**
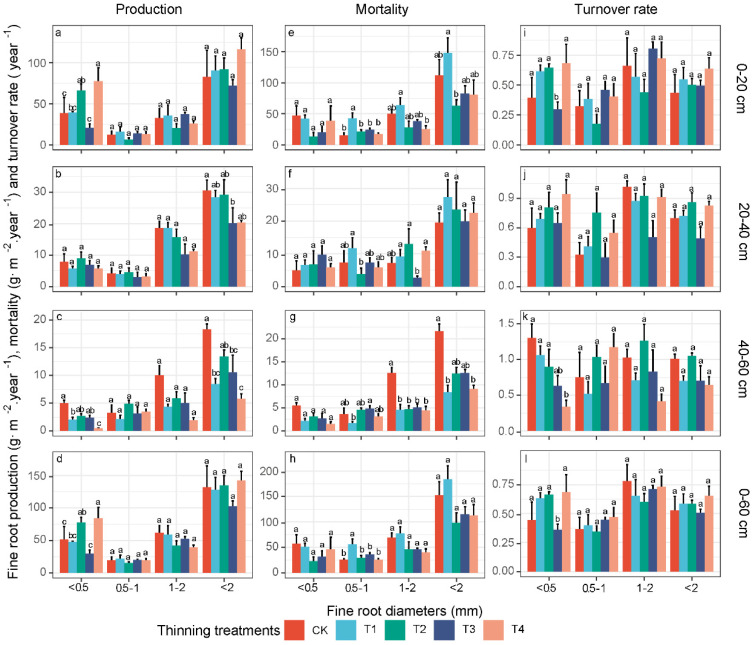
The effects of the thinning intensities on the fine-root production, mortality and turnover rate among different diameter classes at different soil depths (**a**–**c**,**e**–**g**,**i**–**k**), and the entire soil profile (**d**,**h**,**l**). The values are the mean of four replicates ± SE. CK, T1, T2, T3, and T4 represent 0%, 15%, 30%, 45% and 60% thinning intensities, respectively. Different lowercase letters (a, b, c, d) indicate significant differences for the same diameter class of fine roots among the thinning intensities (*p* < 0.05).

The whole fine-root system (Ø ≤ 2 mm) production and mortality of the entire soil profile (0–60 cm) and the averaged turnover rate did not differ among the different thinning intensities (all *p* > 0.05) (Figure 4d,h,l and Table 5). The very fine-root production also fluctuated following thinning, and significantly exceeded that of the CK in T2 and T4 (F = 5.7, *p* < 0.01) (Figure 4d and Table 5). The very fine-root turnover rate in T3 was significantly lower than those at the other thinning intensities (F = 2.71, *p* < 0.05) (Figure 4l and Table 5). The thinning intensities did not affect the production and turnover rates of 0.5–1 mm and 1–2 mm fine roots (all *p* > 0.05) (Figure 4d,l and Table 5). The 0.5–1 mm fine-root mortality value in T1 was higher than that under the other treatments (F = 5.27, *p* < 0.01). The 0.5–1 mm and 1–2 mm fine-root mortality levels mirrored the total mortality levels (Figure 4h and Table 5).

The production and mortality of fine roots decreased with the soil depth, and the values in the topsoil accounted for approximately 69% of the total production and 71% of the total mortality (Figure 4). The production of very fine roots also occupied the topsoil, and accounted for approximately 51% of the topsoil and 35% of the total value. The mortality of 1–2 mm roots was greater at all of the soil depths (Figure 4e–g). The fine-root turnover rate generally increased with deepening soil depths (Figure 4i–k). The very fine-root production and turnover rate were more sensitive to thinning intensities than those of other size classes among the soil depths (Table 5 and Figure 4). The percentage changes in the fine-root production, mortality and turnover rates in the deep soil layers were also generally higher than those in the topsoil following thinning, and these characteristics in the deep soil were enhanced again in T2 (Figure 4 and Table 4).

### 3.4. The Linkages between Fine-Root Characteristics and Stand and Soil Attributes

**Figure 5:**
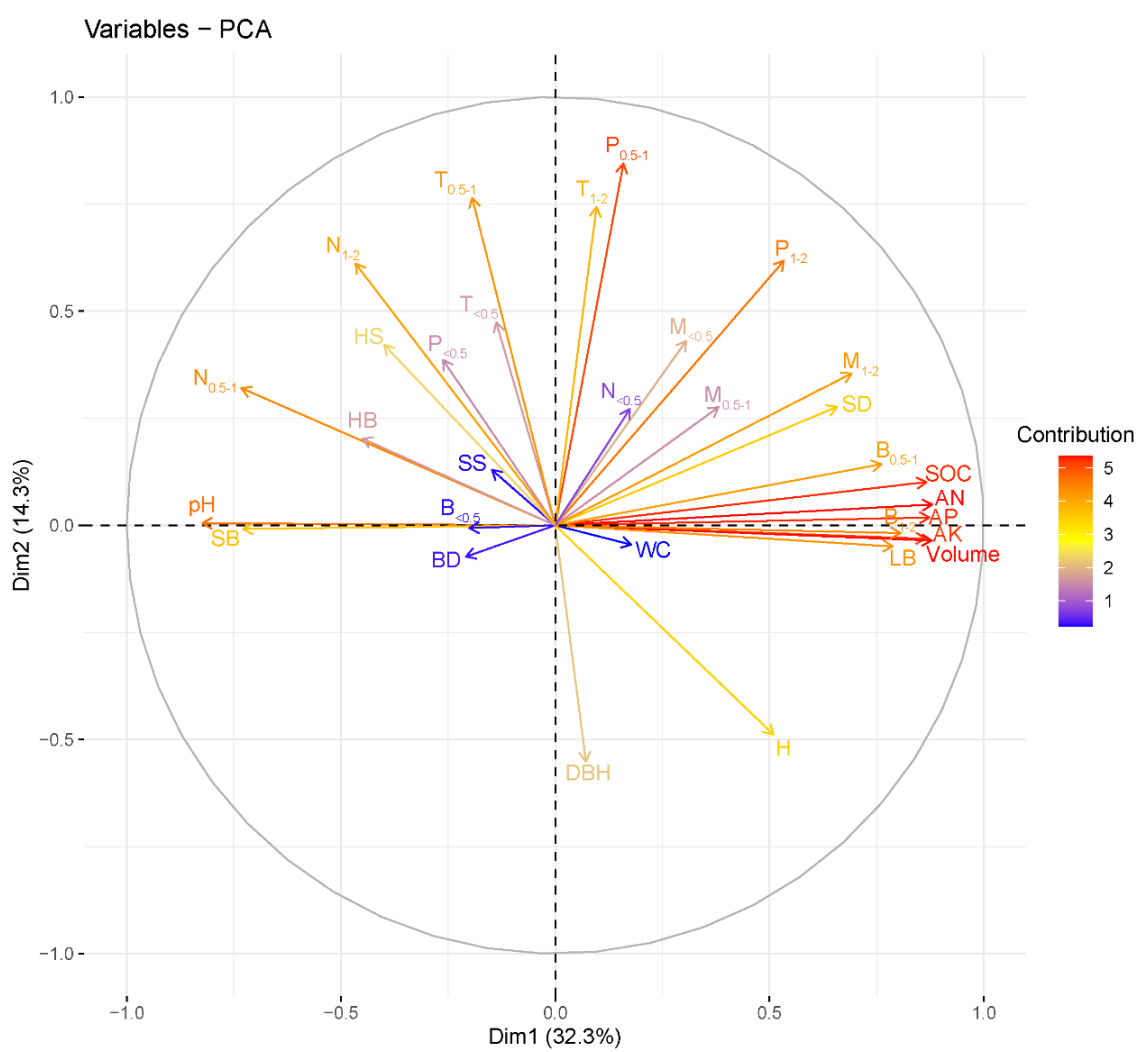
Principal component analysis of the entire soil profile among the fine-root characteristics, stand attributes and soil properties. The arrow of each variable represents the correlation coefficient between the variable and the first two principal components in the unit circle; the variables are colored by their contributions (%) to the variance in the principal component (see the corresponding eigenvalues and eigenvectors in Tables S2 and S3). AN, AP, AK, SOC, WC and BD: the available nitrogen, available phosphorus, available potassium, soil organic carbon, water content, and soil bulk density; DBH, H, SD, SS, HS, SB, HB, LB: diameter at breast height, tree height, stem density, shrub Shannon–Wiener index, herb Shannon–Wiener index, shrub biomass, herb biomass, and litter biomass; P_<0.5_, P_0.5–1_, and P_1–2_: <0.5, 0.5–1, and 1–2 mm fine-root production; M_<0.5_, M_0.5–1_, and M_1–2_: <0.5, 0.5–1, and 1–2 mm fine-root mortality; T_<0.5_, T_0.5–1_, and T_1–2_: <0.5, 0.5–1, and 1–2 mm fine-root turnover rate; B_<0.5_, B_0.5–1_, and B_1–2_: <0.5, 0.5–1, and 1–2 mm fine-root biomass; N_<0.5_, N_0.5–1_, and N_1–2_: <0.5, 0.5–1, and 1–2 mm fine-root necromass.

The PCA of the entire soil profile according to the standardized data shows that the first two trait axes accounted for 32.3% and 14.3% of the total variation, respectively (Figure 5). We found that the 0.5–1 mm and 1–2 mm fine-root biomasses were highly positively related to the decreased soil properties (soil organic carbon, available nitrogen, available phosphorus and available potassium) and the decreased stand characteristics (volume and litter biomass) but were negatively correlated with the increased Shannon–Wiener index and biomass of shrubs and herbs (Figure 5 and Table S4). Conversely, the biomass, production, and turnover rate of very fine roots and the necromass of 0.5–1 mm and 1–2 mm fine roots were negatively correlated with the decreased soil nutrient availability, stand volume and litter biomass, whereas they exhibited strong positive correlations with the increased Shannon–Wiener index and the biomass of shrubs and herbs (Figure 5 and Table S4). The PCA of individual soil depths shows that the association between the understory vegetation characteristics and the very fine-root portion was stronger in the topsoil, whereas this correlation disappeared with the thicker fine-root portion appearing at deeper soil depths (Figure S3).

## 4. Discussion

### 4.1. Effects of Thinning on the Fine-Root Biomass, Production and Turnover Rate

As is consistent with our hypothesis, we found that the very fine-root biomass, production and turnover rates were more sensitive to thinning intensities than thicker fine roots. The results align with a recent observation that forest cutting has more significant effects on fine roots than on thicker roots [18]. These results may be attributed to the fact that fine roots of different diameter classes exhibit heterogeneous physiological functions and structural compositions, leading to discrepant responses following thinning intensities [21]. Moreover, a similar study found that very fine roots with a higher ratio of nonstructural to structural mass are more sensitive to changes in abiotic or biotic factors caused by thinning treatments [24].

The very fine-root biomass, production and turnover rate levels fluctuated (positive or negative effects) with increasing thinning intensities, which is inconsistent with the positive or negative results obtained following thinning in previous studies [3,32]. Regarding positive effects, our PCA results suggest that the increase in the biomass and productivity of very fine roots resulted from increases in herb and shrub layer species diversity and biomass (especially in the topsoil) (Figure 5 and Figure S3a), which compensated for decreased fine-root biomass and production resulting from the cutting of canopy trees, as is consistent with early studies [14,31]. In addition, previous research has demonstrated that low-nutrient conditions can stimulate the growth of fine-root biomass and productivity [51]. In the present study, our whole-tree harvesting measures (reduced stand volume and liter biomass) increased the export of nutrients and reduced soil nutrient availability (Table 2), as is consistent with an early study [52]. Furthermore, the PCA results show that very fine-root biomass and production were negatively correlated with soil nutrient availability, stand volume and litter biomass, supporting this view. Regarding negative effects, a possible explanation is that thinning reduces resource competition pressure, and low fine-root biomass, production and turnover rate values may satisfy vegetation resource absorption and utilization requirements [53].

In contrast, we found that higher thinning intensities significantly reduced the biomass of 0.5–1 mm and 1–2 mm fine roots, supporting the negative effect of thinning found in previous studies [54]. On the one hand, our PCA results indicate that thinning reduced the stand volume and litter biomass, which may decrease the photosynthate partitioning to the root system, and may ultimately lead to the reduced biomass and productivity of 0.5–1 mm and 1–2 mm fine roots [18]. Early studies reported that the nutrient acquisition strategy for thicker fine roots may be achieved by increasing their lifespan and extending the period of nutrient absorption, which is strongly dependent on nutrient availability [20,55,56]. Our PCA results show that 0.5–1 mm and 1–2 mm fine-root biomass was positively correlated with soil nutrient availability, and negatively correlated with herb and shrub species diversity and biomass. Therefore, the decreased stand volume, litter biomass and nutrient availability following thinning together reduced the 0.5–1 mm and 1–2 mm fine-root biomass. Overall, our findings provide a reasonable explanation for the inconsistent impacts of thinning on fine-root biomass and production observed in previous studies [3,32].

In terms of fine-root biomass and productivity, our results reveal that a 30% approach (T2: moderately reduced aboveground biomass) may be a more suitable thinning strategy to promote productivity and increase stand heterogeneity in forest ecosystems. Previous studies have also reported that root gaps in forests recover faster and are more ephemeral than canopy gaps after thinning [57,58], demonstrating that fine-root characteristics serve as very valuable data for the selection of a suitable thinning intensity. Moreover, our findings align with traditional studies showing that a moderate thinning intensity could better improve stand productivity and increase the diversity of understory plants compared to other thinning intensities [59,60].

### 4.2. Necromass and Mortality Changes following Thinning

In this study, the thinning intensity had no influence on the fine-root necromass of the entire soil profile (except for T4). Our observed characteristics of the fine-root necromass are inconsistent with the results of Wang et al. (2019) and Ma et al. (2013) [14], who found thinning intensities to decrease or increase necromass. On the one hand, previous studies have demonstrated that the biomass and necromass of very fine roots account for a greater proportion of the fine-root system than other root size classes [24], and our very fine-root biomass results support this conclusion, which may theoretically determine the necromass. However, very fine roots also possess higher nonstructural carbohydrate concentrations and decompose more easily; thus, these roots are rarely observed [44,61,62]. On the other hand, higher thinning intensities may improve the soil conditions (e.g., soil temperature, Figure S4), and may also further increase the fine-root decomposition rate [32]. Thus, the fine-root necromass levels did not differ between the treatments, potentially due to a large amount of fine-root necromass decomposing and disappearing [63,64]. We note, however, that T4 exhibited significantly increased necromass levels. This finding may be attributable to the fact that high-intensity thinning and species replacement produced a large amount of fine-root necromass [28,30], especially for 0.5–1 mm and 1–2 mm fine roots, which had not completely decomposed, resulting in a significant increase in necromass in T4.

### 4.3. Response of Deeper-Soil Fine-Root Characteristics to Thinning

As hypothesized, we found the thinning effects on the change in the fine-root characteristics to be stronger at deep soil depths, and the fine-root characteristics exhibited fluctuating patterns. These findings are consistent with the results of a recent study showing dramatically altered fine-root biomass and necromass levels at deeper soil depths following thinning [18]. This response may reflect the resource acquisition strategy of the root system following thinning. In the topsoil layer, the fine-root systems of shallow root understory plants and the remaining trees quickly colonize and recover in the area liberated by the disturbance; thus, these roots are less affected by thinning intensities [17,65]. It is well known that trees and understories jointly determine the characteristics of roots in the surface soil layer due to belowground niche partitioning, while trees determine such characteristics at deep soil depths (Figure S3) [66,67]. In our study, all of the four thinning intensities reduced the fine-root biomass and productivity at deep soil depths (Figure 2 and Figure 4), which may be because these intensities reduced the fine-root densities of trees, and consequently alleviated resource competition pressure at this depth [68]. Compared to low- and high-intensity thinning at deep soil depths, higher fine-root biomass and productivity levels were observed in the T2 or T3 thinning treatments (Figure 2 and Figure 4). This change could be attributed not only to thinning interventions but also to the regeneration of understory plants [10]. In the T2 or T3 thinning treatments, the faster regeneration of understory plants excessively consumes resources, which results in greater resource competition pressure in the surface soil, and requires trees to adjust their rooting depth and increase their fine-root growth at deeper soil depths with less root competition [14]. Thus, we observed the fluctuating phenomena of the fine-root characteristics in the deep soil layers. However, the fine-root characteristics at deep soil depths could not recover in a short period of time after thinning (Figure 2 and Figure 4), reflecting their more sensitive response to thinning intensities. Moreover, 0.5–1 and 1–2 mm fine roots dominated the fine-root characteristics at the deep soil depths, and exhibited an increased turnover rate, indicating that thicker fine roots could better mirror potential carbon pools at deeper soil depths in forest ecosystems.

Some studies have reported that fine roots can be categorized according to their functions (e.g., the first order of the root system plays a role in absorption) [44,69]. Furthermore, fine-root characteristics may vary among different functional plant groups [14]. Although the current study uses a more nuanced classification method that diverges from the traditional definition (≤2 mm) to study the stand-level characteristics of fine roots, future studies could build on root functional approaches and plant functional group distinctions in order to better understand how root function specificity and species diversity impact belowground processes at the ecosystem level.

## 5. Conclusions

Our study suggests that different thinning intensities have substantial effects on the changed characteristics of fine roots with different diameters at different soil depths. The positive effect of thinning on very fine roots and the negative effect on thicker fine roots and all of the diameter classes of fine roots provide reasonable explanations for the inconsistent effects of thinning intensities. Here, a 30% (T2) thinning intensity moderately reduced the aboveground biomass and yielded increased biomass and productivity among very fine roots compared to the other treatments, suggesting that a 30% approach is a more suitable thinning strategy to promote productivity and increase stand heterogeneity in forest ecosystems. The fine-root characteristics at deeper soil depths are more sensitive to thinning intensities. The sampled 0.5–1 and 1–2 mm fine roots dominated the fine-root characteristics at deep soil layers and exhibited a higher turnover rate, indicating that thicker fine roots could better mirror the potential carbon pools of deeper soils in forest ecosystems. Collectively, our findings provide important insights into the effects of forest management on change in fine-root characteristics, and supplement meaningful data on fine-root productivity to improve the parameterization of future ecological models.

## Figures and Tables

**Table 1:** Simplified decision matrix for the calculation of the production and mortality of the fine roots.

If	Fine-Root Production	Fine-Root Mortality
Δ*L* + Δ*D* ≥ 0 and Δ*D* ≥ 0	Δ*L* + Δ*D*	Δ*D*
Δ*L* ≥ 0 and Δ*D* ≤ 0	Δ*L*	0
Δ*L* ≤ 0 and Δ*D* ≤ 0	0	|Δ*L*|

Note—L: live fine-root mass; D: dead fine-root mass; Δ represents the changes in living fine-root biomass or necromass. The vertical bars indicate the absolute values. The inequalities in the first column on the left indicate the conditions of the change value of fine-root biomass and necromass.

**Table 2:** Characteristics of the stand and soil (mean ± SE; *n* = 4).

Characteristic	Depth (cm)	CK	T1	T2	T3	T4	*p*
Tree		0%	15%	30%	40%	60%	
Stem density (trees ha^−1^)		1306 ± 158 a	1006 ± 128 ab	845 ± 148 b	614 ± 83 b	595 ± 161 b	<0.001
DBH (cm)		16.77 ± 1.07	18.41 ± 1.33	17.74 ± 1.13	17.06 ± 1.16	17.31 ± 0.74	0.35
Tree height (m)		14.03 ± 0.67	14.72 ± 0.97	13.8 ± 0.61	13.15 ± 0.63	12.59 ± 0.35	0.16
Volume (m^3^ ha^−1^)		250.5 ± 21.56 a	240.08 ± 10.68 a	167.33 ± 17.14 b	117.42 ± 4.08 bc	105.72 ± 16.87 c	<0.001
Understory plants							
Shrub biomass (t ha^−1^)		3.41 ± 0.06 c	3.92 ± 0.23 bc	4.45 ± 0.16 ab	4.94 ± 0.21 ab	4.97 ± 0.58 a	<0.01
Herb biomass (t ha^−1^)		0.62 ± 0.04 b	0.79 ± 0.12 ab	0.87 ± 0.13 ab	1.02 ± 0.12 a	1.02 ± 0.18 a	<0.001
Litter biomass (t ha^−1^)		3.6 ± 0.18 a	2.93 ± 0.28 ab	2.78 ± 0.31 b	2.29 ± 0.27 bc	1.9 ± 0.17 c	<0.001
Shannon-Wiener–herb		1.96 ± 0.05 b	2.13 ± 0.08 ab	2.28 ± 0.13 ab	2.22 ± 0.13 ab	2.39 ± 0.15 a	<0.05
Shannon-Wiener–shrub		1.84 ± 0.09 b	1.91 ± 0.17 b	2.25 ± 0.03 a	2.01 ± 0.05 b	2.19 ± 0.13 ab	<0.05
Soil							
Water content (%)	0–20	44.25 ± 3.08	36.01 ± 2.6	33.13 ± 3.61	44.25 ± 3.74	34.44 ± 1.5	0.051
	20–40	33.22 ± 2.79	29.17 ± 1.42	30.58 ± 2.32	30.46 ± 1.65	27.48 ± 2.2	0.44
	40–60	29.04 ± 1.1	23.3 ± 1.13	25.9 ± 1.59	26.86 ± 1.56	28.41 ± 2.61	0.18
Bulk density (g cm^−3^)	0–20	1.1 ± 0.05	1.26 ± 0.05	1.29 ± 0.11	1.1 ± 0.05	1.31 ± 0.04	0.076
	20–40	1.33 ± 0.05	1.41 ± 0.05	1.41 ± 0.05	1.4 ± 0.05	1.44 ± 0.06	0.63
	40–60	1.41 ± 0.05	1.56 ± 0.03	1.49 ± 0.05	1.48 ± 0.04	1.47 ± 0.07	0.36
SOC (g kg^−1^)	0–20	26.64 ± 5.08 a	23.83 ± 3.18 a	9.22 ± 0.47 b	9.54 ± 1.59 b	7.37 ± 0.55 b	<0.001
	20–40	14.47 ± 1.05 a	15.82 ± 1.05 a	7.65 ± 0.47 b	5.53 ± 0.67 b	5.76 ± 0.45 b	<0.001
	40–60	13.34 ± 0.78 a	8.75 ± 1.15 b	5.08 ± 0.52 c	4.48 ± 0.37 c	3.77 ± 0.2 c	<0.001
AN (mg kg^−1^)	0–20	35.56 ± 2.65 a	29.37 ± 3.93 a	10.07 ± 0.98 b	9.14 ± 0.42 b	7.96 ± 0.4 b	<0.001
	20–40	16.64 ± 1.58 a	16.01 ± 1.38 a	7.97 ± 0.14 b	6.95 ± 0.6 b	6.37 ± 0.18 b	<0.001
	40–60	9.37 ± 1.33 a	7.59 ± 0.94 ab	7.19 ± 0.32 ab	5.52 ± 0.36 bc	4.95 ± 0.12 c	<0.01
AP (mg kg^−1^)	0–20	4.38 ± 0.74 a	3.58 ± 0.39 ab	3.15 ± 0.41 ab	1.84 ± 0.28 b	2.16 ± 0.3 b	<0.01
	20–40	2.46 ± 0.29 ab	3.15 ± 0.54 a	1.95 ± 0.33 ab	1.36 ± 0.16 b	1.18 ± 0.18 b	<0.01
	40–60	1.86 ± 0.24 a	1.78 ± 0.08 a	1.12 ± 0.06 b	0.73 ± 0.04 c	0.71 ± 0.1 c	<0.001
AK (mg kg^−1^)	0–20	156.19 ± 11.78 ab	185.2 ± 7.26 a	134.78 ± 12.64 b	146.29 ± 5.87 ab	110.81 ± 12.08 b	<0.01
	20–40	110.49 ± 6.98 a	97.33 ± 9.32 ab	101.85 ± 6.63 ab	80.37 ± 8.67 ab	77.15 ± 7.71 b	<0.05
	40–60	91.69 ± 12.45 a	96.3 ± 11.96 a	45.27 ± 5.39 b	42.49 ± 6.26 b	43.88 ± 4.38 b	<0.001
pH	0–20	6.29 ± 0.14	6.14 ± 0.11	6.38 ± 0.08	6.42 ± 0.14	6.6 ± 0.07	0.11
	20–40	5.99 ± 0.18	6.02 ± 0.09	6.07 ± 0.17	6.28 ± 0.08	6.37 ± 0.04	0.18
	40–60	5.75 ± 0.09 b	6.11 ± 0.05 a	6.1 ± 0.09 a	6.21 ± 0.07 a	6.37 ± 0.08 a	<0.05

Note—DBH, SOC, AN, AP and AK: diameter at breast height, soil organic carbon, available nitrogen, available phosphorus, and available potassium. CK, T1, T2, T3, and T4 represent 0%, 15%, 30%, 45% and 60% thinning intensities, respectively. Different letters (a, b, c, d) indicate significant differences between the different thinning intensities (*p* < 0.05).

**Table 3:** The effects of the thinning intensity (T) on the mean annual biomass, necromass, and total biomass of fine roots over the entire soil profile, and at specific depths (D).

Characteristic	df	Source	<0.5 mm	0.5–1 mm	1–2 mm	<2 mm
Entire soil profile						
Biomass (g m^−2^)	4	T	10.91 **	4.42 *	9.01 **	2.11
Necromass (g m^−2^)	4	T	2.34	58 **	11.11 **	25.31 **
Total mass (g m^−2^)	4	T	10.62 **	2.33	4.15 *	2.33
Depth-specific response						
Biomass (g m^−2^)	4	T	19.05 **	11.75 **	16.57 **	12.15 **
	2	D	1620 **	894 **	1621 **	3488 **
	8	T × D	9.12 **	6.44 **	6.91 **	12.58 **
Necromass (g m^−2^)	4	T	6.58 **	4.35 **	10.82 **	13.57 **
	2	D	17.74 **	57 **	431 **	580 **
	8	T × D	1.46	2.17 *	6.64 **	8.03 **
Total mass (g m^−2^)	4	T	17 **	6.15 **	9.93 **	8.85 **
	2	D	1558 **	1074 **	2201 **	4109 **
	8	T × D	8.49 **	7.9 **	9.30 **	13.56 **

Note: The F value is used in the table to show the fine roots’ sensitivity difference to the treatment factors. * *p* < 0.05, ** *p* < 0.01.

**Table 4:** Mean percentage changes of the fine-root characteristics within different diameter classes for all of the thinning treatments at different soil depths (mean ± SE; *n* = 4).

Characteristic	Diameter(mm)	0–20 cm	20–40 cm	40–60 cm
Biomass (%)	<0.05	21.44 ± 6.75	32.92 ± 8.26	42.23 ± 10.18
	0.5–1	9.05 ± 2.54	38.02 ± 8.54	14.39 ± 7.43
	1–2	15.14 ± 4.88	17.68 ± 5.01	42.51 ± 4.39
Necromass (%)	<0.05	36.17 ± 13.27	NA	76.42 ± 25.86
	0.5–1	16.51 ± 5.91	27.23 ± 19.99	33.95 ± 11.6
	1–2	20 ± 10.36	46.4 ± 12.96	30.63 ± 7.89
Total mass (%)	<0.05	21.35 ± 6.66	32.38 ± 7.95	41.23 ± 9.84
	0.5–1	7.45 ± 2.72	33.96 ± 7.4	15.06 ± 6.39
	1–2	10.01 ± 1.74	17.34 ± 3.07	41.08 ± 3.01
Production (%)	<0.05	40.04 ± 14.25	19.74 ± 4.09	62.29 ± 9.94
	0.5–1	19.22 ± 5.27	16.52 ± 4.95	23.83 ± 10.96
	1–2	20.62 ± 6.28	25.19 ± 10.41	57.37 ± 8.42
Mortality (%)	<0.05	39.83 ± 14.89	46.03 ± 17.65	57.21 ± 6.48
	0.5–1	36.03 ± 9.42	42.48 ± 7.92	37.38 ± 5.89
	1–2	36.03 ± 6.28	55.04 ± 11.02	62.4 ± 1
Turnover rate (%)	<0.05	48.21 ± 8.83	29.93 ± 11.18	52.15 ± 8.83
	0.5–1	32.98 ± 6.62	59.15 ± 27.89	33.92 ± 9.27
	1–2	19.82 ± 5.21	21.07 ± 9.96	33.33 ± 9.13

Note—NA: no data were detected in the control group.

**Table 5:** The effects of the thinning intensity (T) on the annual production, mortality, and turnover rate of fine roots over the entire soil profile, and at specific depths (D).

Characteristic	df	Source	<0.5 mm	0.5–1 mm	1–2 mm	<2 mm
Entire soil profile						
Production (g m^−2^)	4	T	5.7 **	0.39	1.5	0.75
Mortality (g m^−2^)	4	T	1.32	5.27 **	2.2	2.23
Turnover (year^−1^)	4	T	3.27 *	0.77	0.63	0.63
Depth-specific response						
Production (g m^−2^)	4	T	2.11	0.52	2.12	2.52
	2	D	126.65 **	0.53	40.43 **	153.64 **
	8	T × D	5.49 **	1.05	1.94	2.96 *
Mortality (g m^−2^)	4	T	1.25	1.52	1.62	1.23
	2	D	0.7	47.3 **	125.66 **	123.36 **
	8	T × D	0.82	2.23 *	6.11 **	2.36 *
Turnover (year^−1^)	4	T	1.5	1	0.66	1.93
	2	D	2.97	4.94 *	1.54	8.34 **
	8	T × D	2.63 *	1.26	2	1.77

Note: The F value is used in the table to show the fine roots’ sensitivity difference to the treatment factors in the total soil layer. * *p* < 0.05; ** *p* < 0.01.

## Data Availability

The data are contained within the article or the supplementary figures and supplementary tables.

## Supplementary Materials

The following supporting information can be downloaded at: https://www.mdpi.com/article/10.3390/biology11030351/s1. Figure S1: The effects of the season on the fine-root necromass among different diameter classes in different soil depths and the entire soil profile (mean ± SE, *n* = 4). CK: 0%, T1: 15%, T2: 30%, T3: 45%, T4: 60% of the stand volume removed, respectively. Figure S2: The effects of the season on the fine-root total mass (biomass + necromass) among different diameter classes in different soil depths and entire soil profile (mean ± SE, *n* = 4). CK: 0%, T1: 15%, T2: 30%, T3: 45%, T4: 60% of the stand volume removed, respectively. Figure S3: Principal component analysis between the characteristics of the overstory and understory, and fine-root characteristics in three soil depths. The arrow for each variable is plotted as the correlation coefficient between the variable and the first two principal components in the unit circle; the variables are colored by their contributions (%) to the variance in a given principal component. DBH: diameter at breast height; H: tree height; SD: stem density; SS: shrub Shannon–Wiener index; HS: herb Shannon–Wiener index; SB: shrub biomass; HB: herb biomass; P_<0.5_, P_0.5–1_, and P_1–2_: <0.5, 0.5–1, and 1–2 mm fine-root production; M_<0.5_, M_0.5–1_, and M_1–2_: <0.5, 0.5–1, and 1–2 mm fine-root mortality; T_<0.5_, T_0.5–1_, and T_1–2_: <0.5, 0.5–1, and 1–2 mm fine-root turnover rate; B_<0.5_, B_0.5–1_, and B_1–2_: <0.5, 0.5–1, and 1–2 mm fine-root biomass; N_<0.5_, N_0.5–1_, and N_1–2_: <0.5, 0.5–1, and 1–2 mm fine-root necromass. Figure S4: Temperature under different thinning intensities. These data were measured after the fine-root sampling (autumn in 2019). The values are the mean of four replicates ± SE. CK: 0%, T1: 15%, T2: 30%, T3: 45%, T4: 60% of the stand volume removed, respectively. Different lowercase letters above the bars indicate significant differences among thinning intensities (*p* < 0.05). Table S1: The effects of thinning (T), season (S) and soil depth (D) on the fine-root biomass, necromass, and total biomass. Table S2: Eigenvalues of the entire soil profile from the principal component analysis of the measured values under five thinning intensity treatments in secondary forests. Table S3: First three eigenvectors of the entire soil profile from the PCA of the measured variables under five thinning intensity treatments in secondary forests. Table S4: Coefficients between fine root characteristics and soil properties and stand characteristics.
