# Supplementary material for: Responses of Fine Roots at Different Soil Depths to Different Thinning Intensities in a Secondary Forest in the Qinling Mountains, China
Source: Biology (Basel). 2022 Feb 22;11(3):351. doi: 10.3390/biology11030351 (PMC8945732; doi:10.3390/biology11030351)
Supplement: Supplementary file 1 [file biology-11-00351-s001.zip › biology-1569046-supplementary.pdf]

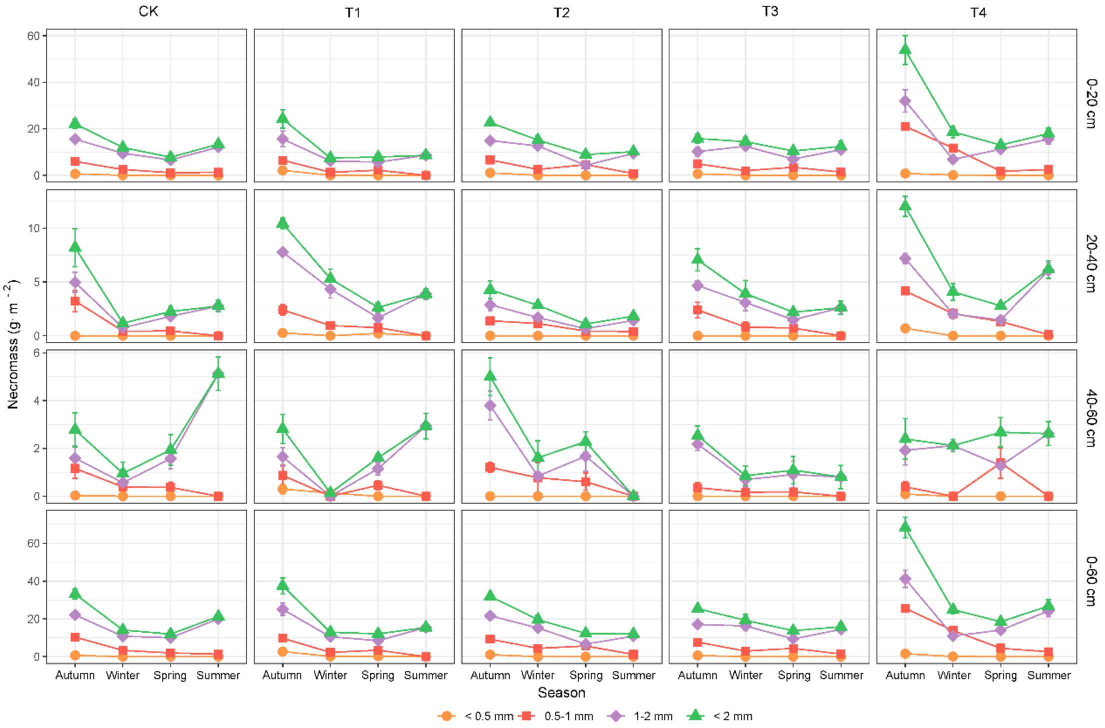

Figure S1. The effects of season on the fine root necromass among different diameter classes in different soil depths and entire soil profile (mean ± SE, n = 4). CK: 0%, T1: 15%, T2: 30%, T3: 45%, T4: 60% of the stand volume removed, respectively.

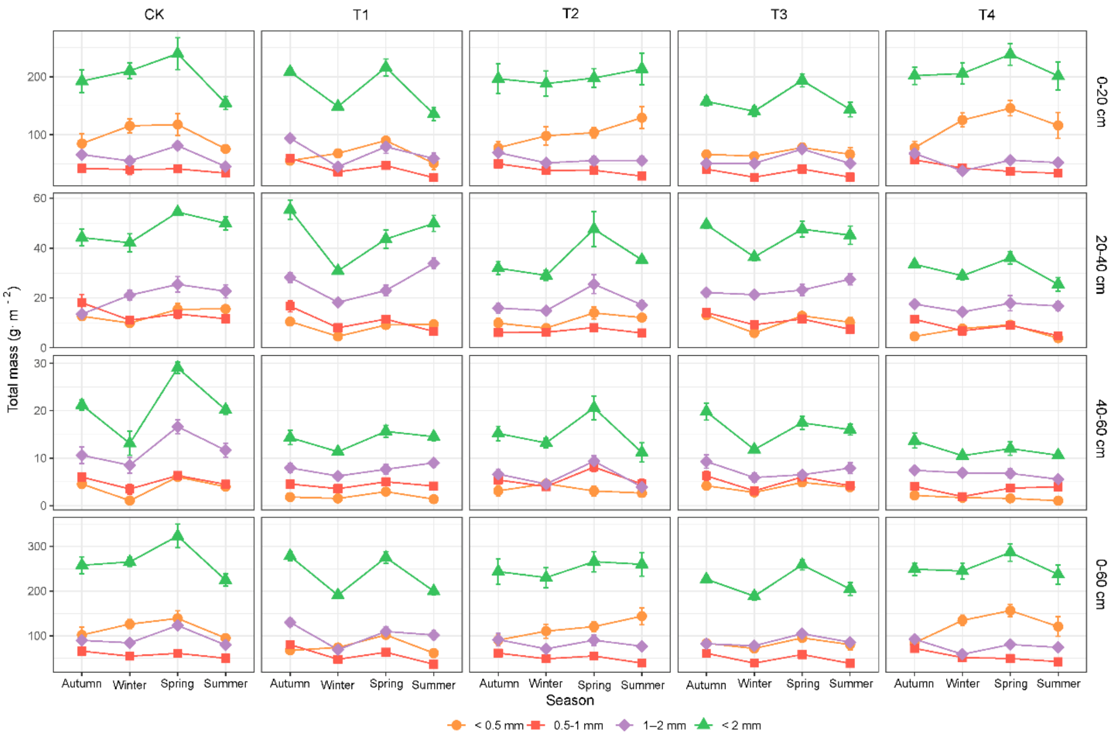

Figure S2. The effects of season on the fine root total mass (biomass + necromass) among different diameter classes in different soil depths and entire soil profile (mean ± SE, n = 4). CK: 0%, T1: 15%, T2: 30%, T3: 45%, T4: 60% of the stand volume removed, respectively.

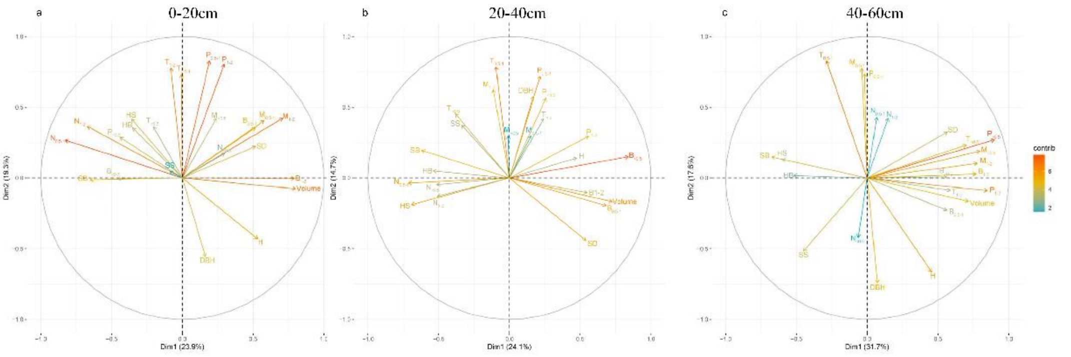

Figure S3. Principal component analysis between characteristics of overstory and understory and fine root dynamics in three soil depths. The arrow for each variable is plotted as the correlation coefficient between the variable and the first two principal components in the unit circle; variables are colored by their contributions (%) to the variance in given principal component. DBH: diameter at breast height; H: tree height; SD: stem density; SS: shrub Shannon-Wiener index; HS: herb Shannon-Wiener index; SB: shrub biomass; HB: herb biomass; $P_{<0.5}$, $P_{0.5\text{-}1}$, $P_{1\text{-}2}$: < 0.5, 0.5-1, 1-2 mm fine root production; $M_{<0.5}$, $M_{0.5\text{-}1}$, $M_{1\text{-}2}$: < 0.5, 0.5-1, 1-2 mm fine root mortality; $T_{<0.5}$, $T_{0.5\text{-}1}$, $T_{1\text{-}2}$: < 0.5, 0.5-1, 1-2 mm fine root turnover rate; $B_{<0.5}$, $B_{0.5\text{-}1}$, $B_{1\text{-}2}$: < 0.5, 0.5-1, 1-2 mm fine root biomass; $N_{<0.5}$, $N_{0.5\text{-}1}$, $N_{1\text{-}2}$: < 0.5, 0.5-1, 1-2 mm fine root necromass.

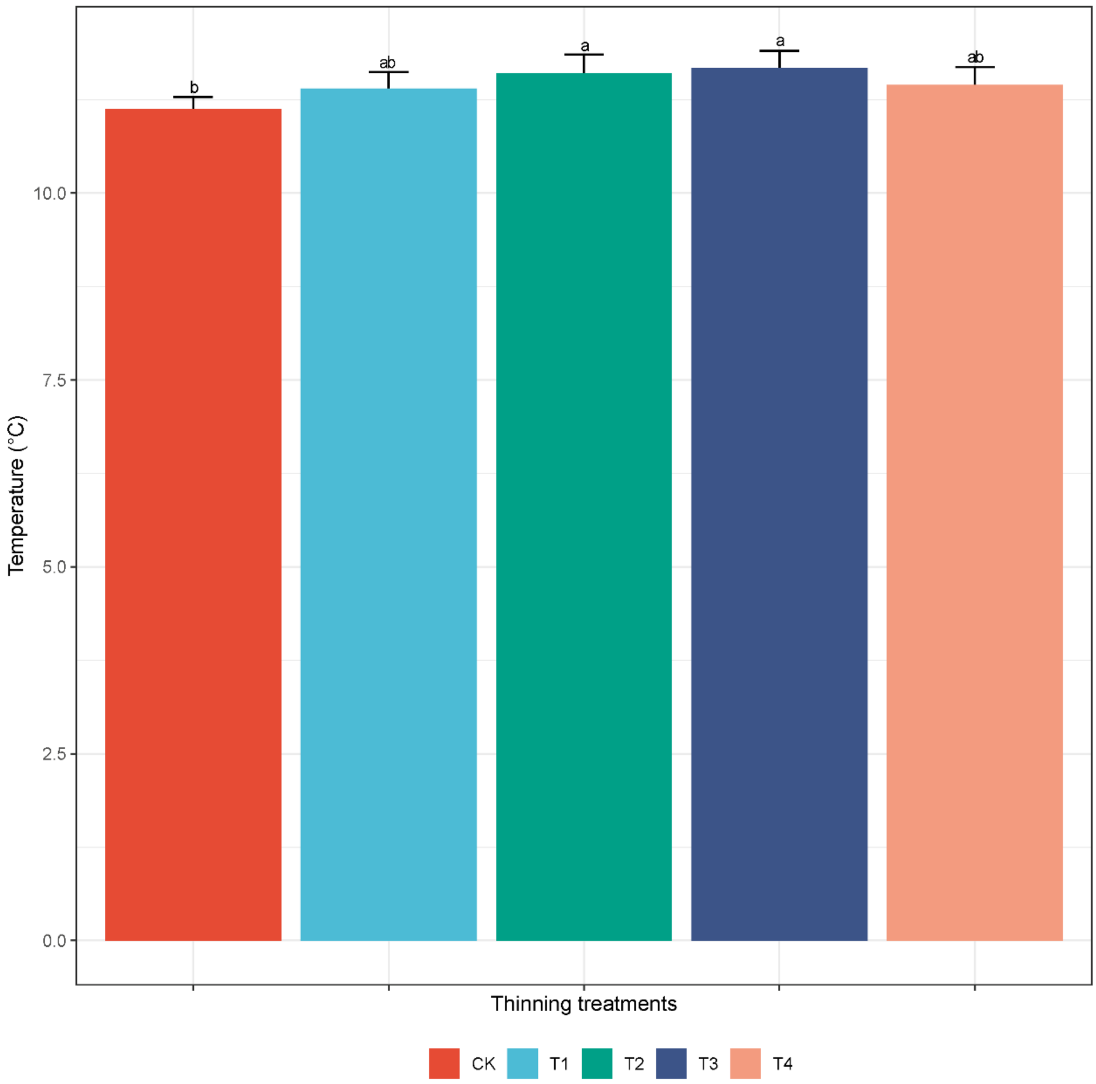

Figure S4. Temperature under different thinning intensities. These data were measured after fine root sampling (autumn in 2019). Values are the mean of 4 replicates ± SE. CK: 0%, T1: 15%, T2: 30%, T3: 45%, T4: 60% of the stand volume removed, respectively. Different lowercase letters above the bars indicate significant differences among thinning intensities ($p < 0.05$).

Table S1. The effects of thinning (T), season (S) and soil depth (D) on the fine root biomass, necromass, and total biomass.

Characteristic	T	S	D	T×S	T×D	S×D	T×S×D
df	4	3	2	12	8	6	24
Biomass (g m^{-2})							
< 0.5 mm	23.81**	15.18**	2194**	4**	11.98**	3.79**	4.03**
0.5-1 mm	15.35**	23.08**	1145**	1.6	8.27**	3.79**	1.48
1-2 mm	17.78**	24.73**	1257**	3.94**	6.03**	5.06**	2.77**
< 2 mm	11.64**	40.09**	4292**	2.89**	14.93**	2.72*	3.7**
Necromass (g m^{-2})							
< 0.5 mm	6.07**	60.75**	12.99**	3.07**	0.94	13.44**	1.4
0.5-1 mm	2.86*	65.29**	89.17**	1.48	1.95	2.93**	2.03**
1-2 mm	7.01**	20.09**	156.56**	7.94**	3.32**	6.5**	6.84**
< 2 mm	8.09**	20.49**	152.27**	6.68**	3.39**	6.16**	6.12**
Total mass (g m^{-2})							
< 0.5 mm	21.27**	14.65**	2141**	3.82**	11.22**	3.84**	3.8**
0.5-1 mm	8.04**	41.36**	1311**	1.32	9.28**	5.63**	1.29
1-2 mm	11.01**	20.47**	1627**	3.62**	7.05**	3.71**	2.61**
< 2 mm	8.41**	39.24**	4601.5**	2.22**	14.46**	3.3**	3.05**

Note: The F value is used in the table to show the fine root's sensitivity difference to treatment factors. *$p < 0.05$, **$p < 0.01$. Linear mixed-effects model fit tests used Satterthwaite approximations for denominator degrees of freedom.

Table S2. Eigenvalues of entire soil profile from the principal component analysis of measured values under five thinning intensity treatments in secondary forests.

**Dimensions**	**Eigenvalue**	**Percentage**	**Accumulated percentage**
**1**	10	32.27	32.27
**2**	4.44	14.33	46.61
**3**	3.65	11.77	58.38
**4**	3.15	10.16	68.54
**5**	2.3	7.43	75.97
**6**	1.67	5.4	81.37
**7**	1.37	4.43	85.8
**8**	0.98	3.15	88.95
**9**	0.89	2.88	91.82
**10**	0.63	2.04	93.87
**11**	0.45	1.46	95.33
**12**	0.44	1.41	96.74
**13**	0.41	1.32	98.06
**14**	0.18	0.59	98.65
**15**	0.16	0.51	99.16
**16**	0.12	0.38	99.54
**17**	0.09	0.28	99.82
**18**	0.04	0.12	99.94
**19**	0.02	0.06	100

Table S3. First three eigenvectors of entire soil profile from the PCA of the measured variables under five thinning intensity treatments in secondary forests.

**Variables**	**Dimensions**		
	**1**	**2**	**3**
**AN**	0.88	0.05	-0.01
**AP**	0.87	0.02	0.22
**AK**	0.86	-0.03	0.08
**SOC**	0.87	0.1	-0.06
**pH**	-0.82	0	0.06
**WC**	0.18	-0.05	-0.75
**BD**	-0.21	-0.07	0.75
**DBH**	0.07	-0.55	0.44
**H**	0.51	-0.49	0.45
**SD**	0.66	0.28	-0.19
**Volume**	0.88	-0.04	0.14
**SS**	-0.15	0.13	0.72
**HS**	-0.4	0.42	0.3
**SB**	-0.73	-0.01	0.14
**HB**	-0.45	0.2	-0.06
**LB**	0.79	-0.05	0.05
$\mathbf{P_{<0.5}}$	-0.26	0.39	0.34
$\mathbf{P_{0.5\text{-}1}}$	0.16	0.84	-0.09
$\mathbf{P_{1\text{-}2}}$	0.53	0.62	-0.23
$\mathbf{M_{<0.5}}$	0.31	0.43	0.23
$\mathbf{M_{0.5\text{-}1}}$	0.38	0.28	0.35
$\mathbf{M_{1\text{-}2}}$	0.69	0.35	0.12
$\mathbf{T_{<0.5}}$	-0.14	0.47	0.42
$\mathbf{T_{0.5\text{-}1}}$	-0.19	0.76	-0.21
$\mathbf{T_{1\text{-}2}}$	0.1	0.74	-0.36
$\mathbf{B_{<0.5}}$	-0.2	-0.01	0.04
$\mathbf{B_{0.5\text{-}1}}$	0.76	0.14	0.2
$\mathbf{B_{1\text{-}2}}$	0.81	-0.02	0.15
$\mathbf{N_{<0.5}}$	0.17	0.27	0.71
$\mathbf{N_{0.5\text{-}1}}$	-0.73	0.32	0.2
$\mathbf{N_{1\text{-}2}}$	-0.47	0.61	0.23

Note: AN: available nitrogen; AP: available phosphorus; AK: available potassium; SOC: soil organic carbon; WC: water content; BD: soil bulk density; DBH: diameter at breast height; H: tree height; SD: stem density; SS: shrub Shannon-Wiener index; HS: herb Shannon-Wiener index; SB: shrub biomass; HB: herb biomass; LB: litter biomass; $P_{<0.5}$, $P_{0.5\text{-}1}$, $P_{1\text{-}2}$: < 0.5, 0.5-1, 1-2 mm fine root production; $M_{<0.5}$, $M_{0.5\text{-}1}$, $M_{1\text{-}2}$: < 0.5, 0.5-1, 1-2 mm fine root mortality; $T_{<0.5}$, $T_{0.5\text{-}1}$, $T_{1\text{-}2}$: < 0.5, 0.5-1, 1-2 mm fine root turnover rate; $B_{<0.5}$, $B_{0.5\text{-}1}$, $B_{1\text{-}2}$: < 0.5, 0.5-1, 1-2 mm fine root biomass; $N_{<0.5}$, $N_{0.5\text{-}1}$, $N_{1\text{-}2}$: < 0.5, 0.5-1, 1-2 mm fine root necromass.

Table S4. Coefficients between fine root characteristics and soil properties and stand characteristics.

Fine root characteristics	Soil properties							Stand characteristics								
	AN	AP	AK	SOC	pH	WC	BD	DBH	H	SD	Volume	SS	HS	SB	HB	LB
$P_{<0.5}$	-0.58	-0.68	-0.54	-0.58	0.01	-0.14	0.02	-0.1	-0.04	0.01	-0.54	0.57	0.59	0.68	0.67	-0.47
$P_{0.5-1}$	0.19	0.14	0.05	0.23	-0.17	-0.05	-0.03	-0.35	-0.33	0.2	0.01	0.04	0.15	-0.14	0.13	-0.02
P_{1-2}	0.39	0.34	0.42	0.4	-0.49	0.12	-0.16	-0.23	-0.12	0.31	0.24	0.16	0.19	-0.33	-0.17	0.41
$M_{<0.5}$	0.2	0.28	0.12	0.21	-0.16	0.09	-0.16	-0.13	0.11	0.47	0.42	0.36	0.11	-0.35	-0.44	0.32
$M_{0.5-1}$	0.26	0.31	0.32	0.32	-0.2	0.25	-0.15	-0.21	0.04	0.05	0.2	-0.23	0.08	-0.1	-0.17	0.02
M_{1-2}	0.48	0.52	0.67	0.48	-0.58	0.04	-0.1	-0.01	0.25	0.35	0.51	-0.04	-0.11	-0.34	0.01	0.53
$T_{<0.5}$	-0.32	-0.45	-0.46	-0.5	0.01	-0.14	0.11	-0.04	-0.05	0.06	-0.52	0.44	0.34	0.27	0.32	-0.28
$T_{0.5-1}$	-0.07	-0.13	-0.27	-0.02	0.14	-0.04	-0.02	-0.4	-0.54	0.05	-0.27	0.05	0.22	0.12	0.13	-0.34
T_{1-2}	0.05	0.02	0.08	0.08	-0.11	-0.07	-0.1	-0.43	-0.41	0.16	-0.1	0.27	0.07	-0.01	0.28	-0.11
$B_{<0.5}$	-0.49	-0.57	-0.62	-0.64	0.03	-0.11	0.14	-0.08	-0.08	0.01	-0.59	0.65	0.47	0.36	0.57	-0.54
$B_{0.5-1}$	0.56	0.56	0.68	0.51	-0.63	0.01	-0.03	0.13	0.48	0.33	0.58	-0.35	-0.27	-0.57	-0.3	0.68
B_{1-2}	0.64	0.66	0.62	0.62	-0.73	0.07	-0.06	0.32	0.4	0.21	0.54	-0.27	-0.3	-0.5	-0.27	0.53
$N_{<0.5}$	0.2	0.42	0.27	0.24	-0.04	0.59	-0.5	-0.01	0.22	0.15	0.3	0.37	0.41	-0.1	-0.12	0.01
$N_{0.5-1}$	-0.48	-0.52	-0.6	-0.49	0.58	-0.18	0.2	-0.11	-0.41	-0.34	-0.55	0.26	0.43	0.38	0.31	-0.55
N_{1-2}	-0.21	-0.24	-0.4	-0.19	0.46	-0.15	0.11	-0.25	-0.47	-0.07	-0.29	0.38	0.48	0.34	0.12	-0.38

Note: AN: available nitrogen; AP: available phosphorus; AK: available potassium; SOC: soil organic carbon; WC: water content; BD: soil bulk density; DBH: diameter at breast height; H: tree height; SD: stem density; SS: shrub Shannon-Wiener index; HS: herb Shannon-Wiener index; SB: shrub biomass; HB: herb biomass; LB: litter biomass; $P_{<0.5}$, $P_{0.5-1}$, P_{1-2}: < 0.5, 0.5-1, 1-2 mm fine root production; $M_{<0.5}$, $M_{0.5-1}$, M_{1-2}: < 0.5, 0.5-1, 1-2 mm fine root mortality; $T_{<0.5}$, $T_{0.5-1}$, T_{1-2}: < 0.5, 0.5-1, 1-2 mm fine root turnover rate; $B_{<0.5}$, $B_{0.5-1}$, B_{1-2}: < 0.5, 0.5-1, 1-2 mm fine root biomass; $N_{<0.5}$, $N_{0.5-1}$, N_{1-2}: < 0.5, 0.5-1, 1-2 mm fine root necromass.
